# Inclisiran: A New Pharmacological Approach for Hypercholesterolemia

**DOI:** 10.31083/j.rcm2311375

**Published:** 2022-11-03

**Authors:** Stefania Angela Di Fusco, Aldo Pietro Maggioni, Chiara Bernelli, Francesco Perone, Vincenzo De Marzo, Edoardo Conte, Francesca Musella, Giuseppe Uccello, Leonardo De Luca, Domenico Gabrielli, Michele Massimo Gulizia, Fabrizio Oliva, Furio Colivicchi

**Affiliations:** ^1^Emergency Department, Clinical and Rehabilitation Cardiology Unit, San Filippo Neri Hospital, ASL Roma 1, 00135 Rome, Italy; ^2^ANMCO Research Center, Heart Care Foundation, 50121 Florence, Italy; ^3^Cardiology Department, Interventional Cardiology Unit, Ospedale Santa Corona, 17027 Pietra Ligure, Italy; ^4^Cardiac Rehabilitation Unit, Rehabilitation Clinic “Villa delle Magnolie", Castel Morrone, 81100 Caserta, Italy; ^5^CardioThoracic and Vascular Department, San Martino Policlinico Hospital, IRCCS for Oncology and Neurosciences, 16100 Genoa, Italy; ^6^Department of Biomedical Sciences for Health, IRCCS Ospedale Galeazzi-Sant'Ambrogio, University of Milan, 20161 Milan, Italy; ^7^Division of Cardiology, Ospedale Santa Maria delle Grazie Pozzuoli, 80078 Napoli, Italy; ^8^Cardiovascula Department, Clinical and Interventional Cardiology Unit, Istituto Clinico Sant'Ambrogio, 20149 Milan, Italy; ^9^Dipartimento Cardio-Toraco-Vascolare, U.O.C. Cardiologia, Azienda Ospedaliera San Camillo Forlanini, 00152 Roma, Italy; ^10^U.O.C. Cardiologia, Ospedale Garibaldi-Nesima, Azienda di Rilievo Nazionale e Alta Specializzazione “Garibaldi", 95122 Catania, Italy; ^11^De Gasperis Cardio Center, Niguarda Hospital, 20162 Milano, Italy

**Keywords:** hypercholesterolemia, LDL-cholesterol, inclisiran, siRNA, cardiovascular disease

## Abstract

Therapeutic approaches based on gene silencing technologies represent a new opportunity to manage hypercholesterolemia. Inclisiran is a small interfering RNA that targets proprotein convertase subtilisin/kexin type 9 (PCSK9) mRNA. Clinical studies have demonstrated that inclisiran is effective, safe, and well-tolerated in reducing low-density lipoprotein cholesterol (LDL-C) in patients with familial hypercholesterolemia, atherosclerotic cardiovascular disease, and atherosclerotic cardiovascular disease risk equivalents. A meta-analysis of phase 3 trials demonstrated a 51% reduction in LDL-C levels at 18 months as compared with placebo. Adverse event incidence was found to be comparable in individuals treated with inclisiran and those receiving placebo, though the reactions at the site of injection were more common in patients receiving inclisiran as compared with those receiving placebo. The recommended inclisiran dose is 284 mg administered as a subcutaneous injection to be repeated after three months with a subsequent 6-month maintenance regimen. Overall, since the pharmacological efficacy of inclisiran in LDL-C reduction is comparable to that of monoclonal antibodies against PCSK9, the longer effect duration and the favorable safety profile may favor this newer approach for hypercholesterolemia management.

## 1. Lipid-Lowering Therapies: The Need for New Pharmacological Approaches

Dyslipidemia is a modifiable cardiovascular risk factor that contributes to atherosclerosis pathogenesis. Alongside healthy lifestyle interventions, lipid-lowering therapies represent the cornerstone of cardiovascular disease (CVD) risk reduction [1]. It has been estimated that 1 mmol/L (38.67 mg/dL) decrease in low-density lipoprotein cholesterol (LDL-C), the leading cause of atherosclerosis, reduces cardiovascular event incidence by 22% [2]. However, although there has been a progressive growth in the last decade in the therapeutic armamentarium available for reducing LDL-C, substantial CVD risk persists.

According to guidelines, statins represent the first line of therapy. At present, for patients who do not reach LDL-C therapeutic goals, the recommendation is to combine statins with ezetimibe and, if this combination is still insufficient to achieve the desired goal, to add monoclonal antibodies against proprotein convertase subtilisin/kexin type 9 (PCSK9) [3].

Two monoclonal antibodies, evolocumab and alirocumab, are currently available to treat patients at increased risk for CVD. By interfering with PCSK9 binding to the LDL receptor, they reduce the lysosomal breakdown of the LDL receptors in hepatocytes. The final effect is similar to that of statins, leading to LDL receptor upregulation and greater circulating LDL-C clearance. PCSK9 inhibitor monoclonal antibodies lower LDL-C by 50–60% and reduce the risk for CVD proportionally to the LDL-C reduction obtained [4, 5]. A further lipid-lowering strategy with less widespread use in clinical practice includes lipoprotein apheresis [6], a non-pharmacological extracorporeal intervention that is recommended in patients with homozygous or heterozygous (if uncontrolled with drugs) familial hypercholesterolemia.

Despite the different lipid-lowering treatments currently available, there are still several concerns about side effects, costs, and adherence issues that limit optimal lipid control. Observational studies have shown a significant gap between LDL-C levels achieved in clinical practice and the recommended goals [7, 8]. This gap is even greater in patient populations at higher CVD risk [7, 8].

Statins are associated with poor adherence and a high discontinuation rate, and a sizeable share of patients do not attain LDL-C goals. Although statin-induced myopathy risk has been shown to be low, in observational studies statin-associated muscle symptoms are more frequent than those reported in randomized clinical studies (RCTs) and often result in statin therapy discontinuation with a consequent increase in adverse cardiovascular event risk [9]. In large U.S. observational studies, around 10% of patients suspend a statin because of subjective complaints, the most common of which is muscle symptoms [10]. Ezetimibe is generally better tolerated than statins, but it leads to a lower reduction in LDL-C levels. Targeting PCSK9 has recently changed the paradigm of lipid-lowering therapy since it leads to a greater reduction in LDL-C. However, monoclonal antibody PCSK9 inhibitors are currently not used on a large scale mainly because of their high cost.

Innovative therapeutic approaches based on gene silencing technologies represent a new opportunity to manage CVD risk due to dyslipidemia [11]. This new therapeutic strategy uses antisense oligonucleotides (ASOs) or small interfering RNAs (siRNAs) for silencing gene expression. ASOs are nucleotide single-stranded chains that combine with their target RNA, leading to its degradation and thereby determining reduced protein synthesis. siRNA molecules are double-stranded sequences whose antisense strand, once inside the cell, binds to its target mRNA sequence and inhibits protein synthesis by inducing its cleavage. Inclisiran is a new lipid-lowering agent in a late stage of development that acts by silencing PCSK9 gene expression in the liver cells, therefore acting upstream with respect to monoclonal antibodies that antagonize circulating PCSK9 activity. This novel approach could fill the gap in achieving LDL-C target levels in patients at increased risk for CVD. Indeed, although there are still no outcome results for inclisiran, the clinical use of this new lipid-lowering agent has been recently approved in several countries.

This paper discusses available evidence on this new therapeutic option. We briefly illustrate its mechanism of action and report available data on its efficacy and safety. Finally, we discuss its current therapeutic applications.

## 2. Focus on Structure, Mechanism of Action, and Pharmacokinetics

Inclisiran molecule is a synthetic RNA whose target is the mRNA that encodes PCSK9. In order to prevent rapid siRNA molecule degradation in the bloodstream, some chemical modifications, including 20-O-methyl and 20-fluoro nucleotides and some phosphorothioate linkages that are used in substitution of phosphodiester linkages, are introduced.

Furthermore, to direct siRNA uptake within hepatocytes and avoid unintended effects on other cells, the sense strand is conjugated with N-acetylgalactosamine (GalNAc), which combines with the asialoglycoprotein receptor present on the surface of the liver cells. The asialoglycoprotein receptor in turn mediates inclisiran endocytosis. The stability of siRNA molecules stored in intracitoplasmatic compartments, which act as intracell drug depots, may explain the persistence of inclisiran action over time [12].

**Fig. 1.**
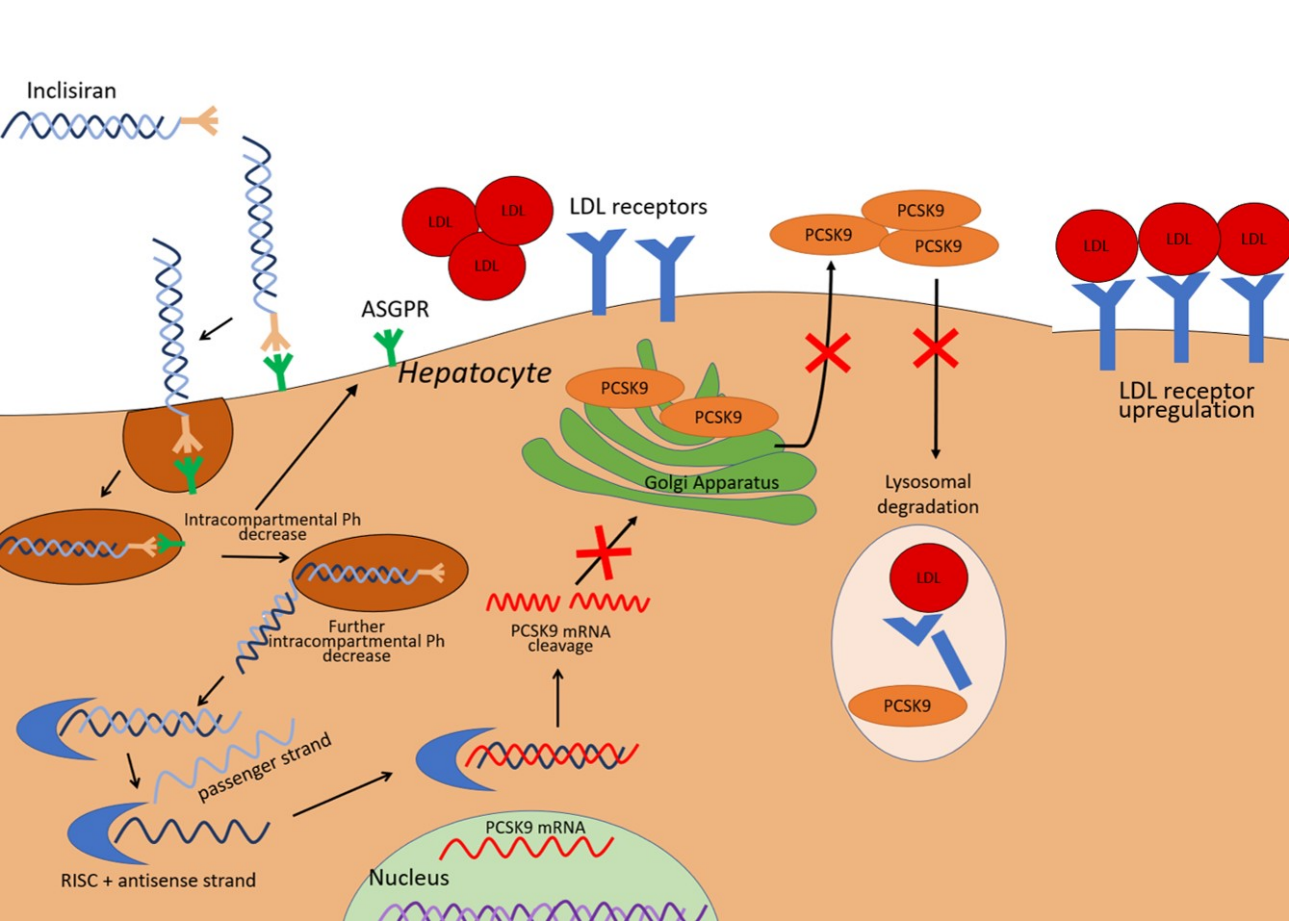
**Illustration of inclisiran effects on the liver cell**. PCSK9 expressed by the liver mediates LDL-receptor degradation in lysosomes. Inclisiran is a small interfering RNA able to promote the degradation of the mRNA that encodes PCSK9, thereby it reduces PCSK9 availability and leads to an increased expression of LDL receptors. The result is an enhanced LDL-C clearance. ASGPR, asialoglycoprotein receptor; LDL-C, low-density lipoprotein cholesterol; PCSK9, proprotein convertase subtilisin/kexin type 9; RISC, RNA-induced silencing complex; mRNA, messenger RNA.

Inclisiran is composed of two complementary RNA chains, an antisense strand (also referred to as a guide strand) and a sense strand (also referred to as a passenger strand). The passenger strand allows siRNA to be integrated into the RNA-induced silencing complex and therefore dissociate from the antisense strand, which is then free to bind mRNA for PCSK9 (Fig. 1). The mRNA target is subsequently cleaved by argonaute-2, a protein component of the RNA-induced silencing complex (RISC). The result is the prevention of mRNA translation in PCSK9. A 66–74% reduction in circulating PCSK9 levels has been reported at 30 days after a single dose, with a similar magnitude of reduction persistent at 60 and 90 days [13]. Since PCSK9 has a central role in the recycling of LDL receptors, the consequence of lower PCSK9 hepatic expression is reduced LDL-receptor degradation and increased LDL particle clearance by hepatocytes, and thus a decrease in circulating LDL-C. Fourteen days after the first administration of inclisiran, a decrease in LDL-C levels of around 40% has been observed. After 30 days, the mean decrease in LDL-C was 45–51%. Reductions in nadir, ranging between 50 and 60%, were observed at 150 days with the two-dose regimen [13].

After subcutaneous administration, inclisiran nadir plasmatic concentration is reached in 4 hours. It is subsequently quickly cleared from plasma by hepatocytes and after 48 hours it is undetectable in the bloodstream [14]. Like other siRNAs, inclisiran is characterized by a temporal pharmacokinetic and pharmacodynamic discrepancy with short duration plasma drug exposure (hours) and persistence of pharmacodynamic effects in the target cells for a long time (months) due to its storage in intracellular compartments.

Of note, even if PCSK9 inhibition does not reduce C reactive protein levels in clinical trials, pre-clinical and clinical studies suggest a potential role of PCSK9 in inflammation processes [15]. It has been demonstrated that PCSK9 may interact with receptors other than LDL receptors, including those that are expressed on macrophage cell surfaces and vascular smooth muscle cells, to further impact atherogenesis.

Kidney disease does not impact inclisiran safety or efficacy. Comparable effects in terms of LDL-C reductions have been reported in all renal disease states [16]. In mild or moderate liver dysfunction, the drug safety profile is comparable to that found in patients with normal liver function [17]. However, individuals with moderate liver dysfunction seem to have a lower percentage change in LDL-C. No data are available about inclisiran efficacy and safety in patients with severe hepatic impairment. In clinical practice, no dose adjustment is needed in patients with kidney disease or in those with mild-to-moderate liver dysfunction, while cautious use is recommended in severe liver dysfunction [16].

## 3. Clinical Evidence

In the first preclinical studies (Table 1, Ref [18, 19]), siRNAs targeting PCSK9 mRNA were formulated in lipid nanoparticles. When tested in rodents and non-human primates, these agents led to a decrease in circulating PCSK9 by 50–70% and in LDL-C levels up to 60% [18]. The effects were dose dependent and were confirmed in a phase 1 study [20]. These first anti-PCSK9 siRNAs were not chemically modified or conjugated to GalNAc and were included in lipidoid nanoparticles to facilitate liver delivery. However, these formulations needed intravenous administration. This limitation led to lipidoid formulations being abandoned and to the development of inclisiran, an siRNA administered by subcutaneous injections. To increase molecule stability in the bloodstream and reduce the risk of RNA strand degradation by circulating nucleases, some structural modification to siRNA molecules, as described in the previous section, have been introduced. Furthermore, the development of siRNA molecules conjugated with GalNAc allowed oligonucleotide uptake to be directed within hepatocytes to avoid unintended effects on other cells [19]. In preclinical studies, inclisiran was tested in non-human primates and led to a substantial decrease in PCSK9 (>80%) and LDL-C (~60%) levels [19].

**Table 1.** **Preclinical studies on siRNA targeting PCSK9 mRNA**.

Study, year	Experimental models	Design	Main Findings
Frank-Kamenetsky *et al*., 2008 [18]	Mice and rats	Different doses of the lipidoid-formulated siRNA molecule were intravenously injected into mice and rats.	siRNA displayed a dose-response decrease in PCSK9 levels.
			Maximal PCSK9 mRNA silencing of 50–70% at a dose of 5 mg/kg.
			The reduction in mRNA transcription translated into a 30% (for mice) and 60% (for rats) reduction in total plasma cholesterol.
	Non-human primates	Animals were randomized to receive a 5 mg/kg dose of si-RNA molecule or to serve as controls	Inclisiran 5 mg/kg administered as a single dose resulted in a statistically significant decrease in LDL-C starting from day 3.
			LDL-C levels returned to basal values after 14–21 days.
			A significant reduction in plasma PCSK9 concentration was observed.
Unpublished data reported by Fitzgerald *et al*., 2017 [19]	Non-human primates	Different doses of siRNA (inclisiran) subcutaneously administered to NHPs	With doses greater than 3mg/kg a substantial decrease in PCSK9 (>80%) and LDL-C (∼60%) was reported, with nadir effects lasting >30 days and a return to basal levels was observed after 90–120 days

LDL-C, low-density lipoprotein cholesterol; NHP, non-human primates; PCSK9, proprotein convertase subtilisin/kexin type 9; siRNA, small interfering RNA.

In clinical studies, inclisiran has been shown to be safe and effective in inducing long-lasting reductions in PCSK9 and LDL-C circulating levels (Table 2, Ref [13, 19, 21, 22, 23, 24]).

**Table 2.** **Clinical studies investigating inclisiran effect on lipid parameters**.

Study (year) [NCT registration number]	Number of patients	Population	Design	Study strategy (intervention duration)	Therapeutic regimen	Study start/ completion dates	Key findings
Fitzgerald *et al*., 2017 [19] [NCT02314442]	69	Healthy individuals with LDL-C ≥100 mg/dL ± statin therapy.	RCT, single blind	Patients 3:1 randomly assigned to inclisiran or placebo (180 days)	Single-increasing-dose (25, 100, 300, 500, or 800 mg) vs a multiple-dose (four doses of 125 mg once a week, followed by two doses of 250 mg once a week, or two doses of 300 or 500 mg once a month)	December 2014/ November 2015	In the single-dose group, inclisiran ≥300 mg decreased PCSK9 levels (up to a 74.5% least-squares mean decrease at day 84 compared to basal values), doses ≥100 mg decreased LDL-C levels (up to a 50.6% least-squares mean decrease compared to basal value). Decreased PCSK9 and LDL-C levels persisted at day 180 for inclisiran doses ≥300 mg. All multiple-dose regimens lowered PCSK9 levels (up to an 83.8% least-squares mean decrease at day 84 compared to basal levels) and LDL-C (up to a 59.7% least-squares mean decrease at day 84 compared to basal levels).
Ray *et al*., 2017 [13] (ORION-1, 2017) [NCT02597127]	501	Patients at high risk for ASCVD with high LDL-C levels despite the maximum statin dose ± other lipid-lowering agents.	RCT, double-blind	1:1 randomization to receive inclisiran or placebo (240 days)	Administration of a single dose of placebo vs inclisiran 200, 300, or 500 mg or two doses (at days 1 and 90) of placebo was two doses of inclisiran 100, 200, or 300 mg	January 2016/ June 2017	At day 180, inclisiran single dose reduced LDL-C least-squares mean by 27.9–41.9% and by 35.5–52.6% after two doses. The greatest decrease in LDL-C was obtained with two-dose 300 mg regimen: 48% of patients reached LDL-C <50 mg/dL. At day 240, patients maintained PCSK9 and LDL-C levels lower than basal values with all treatment regimens.
							Serious adverse events were observed in 11% of patients treated with inclisiran and in 8% of patients of the placebo group. Reactions at the site of injection were observed in 5% of patients treated with inclisiran
Ray *et al*., 2018 [21] [NCT02597127]	501	Patients at high risk for ASCVD	Prespecified analysis of an RCT study	1:1 randomization placebo or inclisiran (210 days)	Administration of a single dose of placebo or inclisiran 200, 300, or 500 mg or two doses) of placebo or inclisiran 100, 200, or 300 mg (the first at day 1 and the second at day 90)	January 2016/ June 2017	Inclisiran single dose decreased, non–HDL-C, VLDL-C, and apo B over 210 days. At day 180, a dose-dependent reduction in non–HDL-C was observed: –25% with single-dose of inclisiran 200 mg; –46% with two doses of inclisiran 300 mg. Apo B was also reduced (–23% and –41%) with the above-mentioned dose schemes.
Ray *et al*., 2019 [22] [NCT02597127]	501	Patients with high CVD risk	Prespecified analysis of an RCT study	Follow-up at 1 year of ORION-1 study patients (360 days)	Monthly check of LDL-C and PCSK9 levels.	January 2016/ June 2018	Decrease in LDL-C after inclisiran single dose was 29.5%, 38.7% and 29.9%–46.4% after inclisiran two doses. The 2-dose regimen of inclisiran 300 mg led to the highest rate of responders and the greatest LDL-C decrease over one year.
Raal *et al*., 2020 [23] (ORION-9) [NCT03397121]	482	Patients with heterozygous FH	RCT, double-blind	1:1 randomization to inclisiran or placebo (540 days)	Inclisiran 300 mg subcutaneous injections or placebo at day 1, 90, 270, and 450.	November 2017/ September 2019	At day 510, a 39.7% LDL-C reduction was observed in the inclisiran group, with substantial decrease in LDL-C levels in all FH genotypes.
Ray *et al*., 2020 [24] (ORION-10) [NCT03399370]	1561	Patients with ASCVD and high LDL-C with maximum tolerated statin dose ± other lipid- lowering agents	RCT, double-blind	1:1 randomization to receive inclisiran or placebo (540 days)	Subcutaneous administration of inclisiran 284 mg or placebo at day 1, 90, and every six months thereafter over 540 days	December 2017/ September 2019	At day 510, a 51.3% reduction in LDL-C was observed in the group treated with inclisiran.
Ray *et al*., 2020 [24] (ORION-11) [NCT03400800]	1617	Patients with ASCVD or ASCVD equivalent and high LDL-C despite maximum tolerated statin dose ± other lipid- lowering agents	RCT, double-blind	1:1 randomization to inclisiran or placebo (540 days)	Subcutaneous administration of inclisiran 284 mg or placebo at day 1, 90, and every six months thereafter over a period of 540 days	November 2017/ August 2019	At day 510, a 45.8% LDL-C reduction was observed in patients treated with inclisiran.

ASCVD, atherosclerotic cardiovascular disease; Apo B, apolipoprotein B; CI, confidence interval; CVD, cardiovascular disease; FH, familial hypercholesterolemia; LDL-C, low-density lipoprotein cholesterol; HDL-C, high-density lipoprotein cholesterol; PCSK9, proprotein convertase subtilisin-kexin type 9; RCT, randomized controlled trial; VLDL-C, very-low-density lipoprotein cholesterol.

The ORION-1 phase 2 study was a multicenter RCT that enrolled 501 individuals with an increased risk of CVD and high levels of LDL-C [13]. Participant mean age was 63 years, 35% were women, 69% had established atherosclerotic cardiovascular disease (ASCVD), and 6% had familial hypercholesterolemia (FH) [13]. Seventy-three percent of included patients were treated with statins. Patients were randomly assigned to receive placebo or 200, 300, or 500 mg of inclisiran in one dose or two doses (at day 1 and day 90). After 180 days from the first administration, the LDL-C percentage change with different inclisiran doses was assessed. The greatest reduction in LDL-C level was observed with a two-dose regimen of inclisiran 300 mg, with a 53% reduction (*p *< 0.001). Notably, inclisiran also reduced PCSK9 by 69% (*p *< 0.001), and high-sensitivity C-reactive protein levels by 17% (*p *< 0.05). Serious adverse events were observed in 11% of patients treated with inclisiran and in 8% of patients treated with placebo. Overall, the most part of the adverse events were reactions at the site of injection that occurred in 4% of patients treated with one dose and in 7% of patients treated with two doses of inclisiran. There was no significant difference in global incidence of adverse events among patients treated with inclisiran and those who received placebo [13]. When inclisiran safety and efficacy was evaluated among patients with and without diabetes, inclisiran treatment was found to be associated with a significant decrease in LDL-C levels over the entire study duration irrespective of the presence of diabetes [25]. Furthermore, at day 180 from treatment initiation and over the entire follow-up duration, glycated hemoglobin levels as compared to basal levels did not change. These findings support the use of inclisiran as a feasible option to reduce LDL-C irrespective of concurrent diabetes.

In the ORION-3 study, an open-label extension of the ORION-1 study, inclisiran was associated with a 51% reduction in LDL-C levels at day 210 and a 77% reduction in PCSK9 [26]. A persistent effect of inclisiran 300 mg was observed over about 22 months and the time-averaged reduction in LDL-C was around 60 mg/dL [26]. Over a follow-up of at least 3 years, no safety issues or laboratory test abnormalities, including hepatic and renal function examinations, were found to be associated with the treatment.

In phase 3 RCTs, inclisiran was tested in patients with FH (ORION- 9), ASCVD (ORION-10), and ASCVD or equivalent risk of ASCVD (ORION-11). Patients included in these studies required further reduction in LDL-C despite maximum tolerated statin treatment [23, 24].

ORION-9 was a double-blind RCT that evaluated inclisiran efficacy and safety in patients with heterozygous FH and LDL-C levels ≥100 mg/dL despite the maximum tolerated dose of lipid-lowering therapies [23]. In this trial, 300 mg of inclisiran sodium salt, equivalent to inclisiran 284 mg, was administered at baseline, after 3 months and then every 6 months, on top of the maximum tolerated dose of lipid-lowering treatments [23]. After 510 days, inclisiran produced a 48% placebo-corrected decrease in LDL-C levels (*p *< 0.0001) and showed a time-adjusted percentage LDL-C reduction of 44% (*p *< 0.0001) from day 90 to day 540 [23].

The ORION-10 and the ORION-11 studies were conducted in the U.S. and in Europe/South Africa, respectively [24]. The first trial enrolled patients with ASCVD and LDL-C levels ≥70 mg/dL. The second trial enrolled patients with ASCVD and LDL-C levels ≥70 mg/dL despite treatment with the maximum tolerated statin dose, or with an ASCVD equivalent risk (type 2 diabetes, FH, or a 10–year cardiovascular risk ≥20% as evaluated by the Framingham Risk Score or equivalent) and LDL-C ≥100 mg/dL [24]. The objectives of both trials were to evaluate inclisiran efficacy, safety, and adverse event incidence over 18 months. Participants were randomized 1:1 to receive inclisiran (284 mg) or placebo. The drug was administered at baseline, after 90 days, and every 6 months thereafter over a period of 540 days. In both trials, the coprimary endpoints were the placebo-adjusted percentage variation in LDL-C from basal levels to day 510 and the time-corrected percentage variation in LDL-C from basal levels to day 90 and 540. The number of patients that were randomized in the ORION-10 and ORION-11 trials was 1561 and 1617, respectively. Stable doses of statins were used in 89.2% of participants in the ORION-10 trial and 94.7% in the ORION-11. At day 510, LDL-C reduction associated with inclisiran was 52% in the ORION-10 trial and 50% in the ORION-11 trial, with a 54% and 49% time-corrected LDL-C decrease, respectively (*p *< 0.001 compared to placebo). In addition, inclisiran lowered PCSK9 levels by 70% in the ORION-10 trial and by 63% in the ORION-11 trial (*p *< 0.001 compared to placebo). Moreover, inclisiran also reduced total cholesterol, apolipoprotein B (ApoB), non-high density lipoprotein cholesterol (non-HDL-C), triglycerides, and lipoprotein (a) when compared to placebo, while inclisiran enhanced high-density lipoprotein cholesterol by 5.1% in ORION-10 and by 6.1% in ORION-11. Adverse event incidence was comparable among patients treated with inclisiran and those treated with placebo in both studies. However, injection site reactions were more common with inclisiran treatment than with placebo. These reactions were generally mild and transient. Of note, in both studies there were no substantial differences in diabetes mellitus incidence between those treated with inclisiran and those treated with placebo [24].

ORION-4 is a double-blind, phase 3 RCT [ClinicalTrials.gov Identifier: NCT03705234] that aims to investigate inclisiran impact on clinical outcomes in patients with ASCVD. Around 15,000 ASCVD patients aged ≥55 years will be randomized 1:1 to be treated with inclisiran sodium salt 300 mg or with placebo (administered as a subcutaneous injection at randomization, 3 months, and every 6 months thereafter) and follow-up for a mean period of 5 years [27]. The ORION-4 primary endpoint is the time to the occurrence of a first major adverse cardiovascular event (MACE) including coronary heart disease death, myocardial infarction, fatal or non-fatal ischemic stroke, or urgent coronary revascularization. Secondary endpoints include the number of patients with a composite of coronary heart disease death or myocardial infarction, the number of patients with cardiovascular death, and the number of patients with a MACE among those treated with high-efficacy statins at baseline. The ORION-4 trial will provide evidence on the inclisiran impact on MACE and on overall prognosis in ASCVD patients [28]. ORION-5 is another ongoing phase 3 RCT [ClinicalTrials.gov Identifier: NCT03851705] aimed at assessing the inclisiran safety, tolerability, and efficacy in individuals with homozygous FH [28].

A meta-analysis of data of 3660 patients included in ORION-9, ORION-10, and ORION-11 has shown that inclisiran treatment is associated with a 51% decrease in LDL-C at 18 months (95% confidence interval, 48–53%; *p *< 0.001), as compared with placebo [29]. Inclisiran also significantly decreased total cholesterol, ApoB, and non-HDL-C, respectively by 37%, 41%, and 45% [29]. This meta-analysis also showed that inclisiran treatment was associated with a 24% reduction in MACE. No differences were observed in the occurrence of hepatic dysfunction or in the changes in creatine kinase plasmatic levels between patients treated with inclisiran and those treated with placebo. However, mild reactions at the site of injection were observed more often among patients treated with inclisiran. Overall, the results of this meta-analysis confirm that inclisiran, used in combination with the maximum tolerated statin dose and in association or not with other lipid-lowering drugs, is effective in reducing LDL-C in individuals with FH, ASCVD, or ASCVD risk equivalents, and is well-tolerated [29].

Two post-hoc aggregate analyses were carried out to assess inclisiran efficacy and safety in two subpopulations of ASCVD patients, those with documented cerebrovascular disease (CEVD) and those with polyvascular disease (PVD) [30, 31]. Patients with CEVD were included if they had a previous ischemic stroke and/or a stenosis greater than 70% of the carotid artery documented by angiography or ultrasound and/or previous percutaneous or surgical carotid revascularization. Patients with CEVD treated with inclisiran achieved a 55.2% mean reduction in LDL-C at day 510 compared to placebo (*p *< 0.0001). Patients with PVD were included in this study if they had ASCVD in at least two main branches of the coronary tree, cerebrovascular vascularity, and/or peripheral arterial vascularity. When treated with inclisiran, these patients had a 51.5% mean reduction in LDL-C at day 510 compared to placebo (*p *< 0.0001), with a safety profile similar to placebo.

Overall, available clinical findings support inclisiran use to lower LDL-C levels in patients at increased ASCVD risk, including patients with FH. Of note, currently there are no available data on studies comparing inclisiran treatment with other lipid-lowering treatment options such as ezetimibe, alirocumab, or evolocumab. Ongoing trials will assess inclisiran efficacy and safety in several different clinical settings, the impact on prognosis, and the persistence of LDL-C reduction with longer treatment duration. Indeed, although inclisiran demonstrated excellent lipid-lowering efficacy in phase 3 trials, we are still awaiting data on cardiovascular outcome that will be provided by ongoing studies. On the basis of current evidence, inclisiran clinical effectiveness is expected to be similar to monoclonal antibodies against PCSK9, which have a similar efficacy in reducing LDL-C levels.

## 4. Therapeutic Applications

Although data on cardiovascular outcome effects of lipid-lowering treatment with inclisiran are currently lacking, a significant reduction in cardiovascular event occurrence may be estimated by using the Cholesterol Treatment Trialist Collaboration meta-analyses results, which reported a substantial reduction (–22%) in cardiovascular risk per 38.67 mg/dL (1 mmol/L) decrease in LDL-C with statin use [32]. Due to the consistent LDL-C reduction achieved with inclisiran treatment, the first approval of inclisiran for clinical use was obtained even before the availability of the results of ongoing clinical trials assessing the clinical impact of treatment on cardiovascular outcomes [27, 28]. To date, several national and international drug agencies have approved its use in patients who do not achieve LDL-C therapeutic targets despite lifestyle interventions and the maximum tolerated dose of statins alone or in combination with other lipid-lowering treatments [14, 33, 34]. In clinical practice, inclisiran can be used to treat patients with hypercholesterolemia and statin resistance or intolerance [35]. The recommended inclisiran dose is 284 mg administered with subcutaneous injection. Subcutaneous administration is repeated at 3 months with a subsequent 6–month maintenance regimen. The dosing regimen is one of the main advantages of inclisiran treatment for lowering LDL-C. This regimen is expected to reduce undertreatment due to adherence issues that often affect currently available lipid-lowering therapies with a daily assumption regimen. It is recommended that inclisiran is administered subcutaneously by healthcare professionals, therefore a system of care that incorporates this treatment in the pathway of patients at increased ASCVD risk should be implemented.

No dose adjustment is needed in older patients or in patients with kidney disease or mild-to-moderate liver dysfunction. However, no data are available on its efficacy and safety in patients with end-stage kidney dysfunction or severe hepatic dysfunction. Since it is eliminated through the kidneys, hemodialysis sessions should be avoided in the 72 hours following administration. Unlike with monoclonal antibodies against PCSK9, no induction of antidrug antibodies interfering with drug efficacy or safety has been observed to date with inclisiran [36].

A potential limitation of inclisiran large-scale use is drug cost. It has been estimated that inclisiran used in patients with ASCVD on top of standard of care may be cost-effective for the U.S. healthcare system at annual prices between $6383 and $13,563 if the quality-adjusted life-year willingness to pay threshold is between $50,000 and $150,000 [37]. At the current price of $3250 per dose for uninsured patients in the USA., inclisiran is estimated to have a cost-effectiveness ratio slightly higher than the threshold of $50,000 per quality-adjusted life-year. Overall, the inclisiran treatment cost per year ($6500) is slightly higher than evolocumab and alirocumab ($5850), two lipid-lowering monoclonal antibodies that have the same biological target and similar efficacy. However, based on several siRNA features, including lower manufacturing process costs and easier drug storage and distribution due to its stability at room temperature, a better cost-effectiveness ratio with inclisiran treatment compared to monoclonal antibodies can be expected in the near future.

## 5. Future Directions

Real-word clinical studies will provide information on inclisiran impact in patients with comorbidities that can affect inclisiran efficacy and safety. An additional aspect that still needs to be defined with additional clinical studies is the management of possible side effects that may persist as long as the drug action. However, the reversal of siRNA-induced gene silencing may be possible [38]. Real-world studies with long follow-up are needed to establish inclisiran long-term safety and possible side effects. Moreover, available evidence has been obtained by using inclisiran on top of standard lipid-lowering treatment, and therefore studies investigating inclisiran efficacy in monotherapy are also warranted. Further information critical for delineating tailored optimal lipid-lowering treatments will be provided by directly comparing inclisiran with monoclonal antibodies against PCSK9. A potentially relevant setting for inclisiran use is in younger people with an elevated risk of premature cardiovascular events. This population may benefit from intensive and early lipid-lowering treatment, and thus would be exposed to drug treatment for a longer time. The ongoing ORION-13 and ORION-16 studies (NCT04659863 and NCT04652726, respectively) will assess inclisiran safety and efficacy in adolescents (aged 12–17 years) with homozygous or heterozygous FH and high LDL-C levels already treated with standard lipid-lowering therapy [39]. Finally, the inclisiran-induced lipoprotein(a) reduction observed in phase 3 trials (19–22%) [24] also deserves further investigation to better define the magnitude of this reduction and its clinical impact [40].

## 6. Conclusions

Although CVD approaches with gene silencing therapies are still at an early stage, inclisiran represents a true milestone in this field. Nevertheless, since the pharmacological efficacy of inclisiran in LDL-C lowering seems to be similar to that of monoclonal antibodies against PCSK9, the longer durability of its effect, which allows a less frequent dosing regimen, and its favorable safety profile may favor this newer approach and contribute to increased treatment persistence and adherence and, finally, to LDL-C goal achievement. Furthermore, the infrequent dosing regimen and high drug tolerability make this treatment a potential optimal early and large-scale preventive intervention that is more acceptable than interventions with daily pill intake [41]. With inclisiran treatment, two major challenges of pharmacological therapies may be overcome: adherence and the interindividual variability of drug effect [42, 43]. Physicians should know emergent LDL-C-lowering therapeutic options, the mechanisms of action of these new treatments, and their indications and contraindications. Ongoing studies evaluating the effects of inclisiran on hard cardiovascular endpoints are also expected to confirm the therapeutic effect of this innovative drug.
